# Commonality of Multidrug-Resistant *Klebsiella pneumoniae* ST348 Isolates in Horses and Humans in Portugal

**DOI:** 10.3389/fmicb.2019.01657

**Published:** 2019-07-18

**Authors:** Filipa Trigo da Roza, Natacha Couto, Carla Carneiro, Eva Cunha, Teresa Rosa, Mariana Magalhães, Luís Tavares, Ângela Novais, Luísa Peixe, John W. Rossen, Luís P. Lamas, Manuela Oliveira

**Affiliations:** ^1^CIISA-Centre for Interdisciplinary Research in Animal Health, Faculty of Veterinary Medicine, University of Lisbon, Lisbon, Portugal; ^2^Department of Medical Microbiology and Infection Prevention, University Medical Center Groningen, University of Groningen, Groningen, Netherlands; ^3^UCIBIO/REQUIMTE, Laboratório de Microbiologia, Faculdade de Farmácia, Universidade do Porto, Porto, Portugal

**Keywords:** antimicrobial resistance, ESBL, horse, *Klebsiella pneumoniae*, ST348

## Abstract

Multidrug-resistant (MDR) *Klebsiella pneumoniae* is considered a major global concern by the World Health Organization. Evidence is growing on the importance of circulation of MDR bacterial populations between animals and humans. Horses have been shown to carry commensal isolates of this bacterial species and can act as human MDR bacteria reservoirs. In this study, we characterized an extended-spectrum β-lactamase (ESBL)-producing *K. pneumoniae* sequence type (ST) 348 isolate from a horse, an ST reported for the first time in an animal, using next-generation sequencing. We compared it with six other MDR *K. pneumoniae* ST348 human isolates previously identified in health-care facilities in Portugal using a core genome multi-locus sequence typing approach to evaluate a possible genetic link. The horse isolate was resistant to most of the antimicrobials tested, including 3rd generation cephalosporins, fluoroquinolones, and aminoglycosides, and presented several antimicrobial resistance genes, including *bla*_ESBL_. Twenty-one allele differences were found between the horse isolate and the most similar human isolate, suggesting a recent common ancestor. Other similarities were observed regarding the content on antimicrobial resistance genes, plasmid incompatibility groups, and capsular and somatic antigens. This study illustrates the relevance of the dissemination of MDR strains, and enhances that identification of these types of bacterial strains in both human and veterinary settings is of significant relevance in order to understand and implement combined control strategies for MDR bacteria in animals and humans.

## Introduction

The dissemination of multidrug-resistant (MDR) bacteria is a recognized worldwide problem in both human and veterinary medicine (Abuzaid et al., 2012). Among MDR strains, extended spectrum ß-lactamase (ESBL) producing *Klebsiella pneumoniae* are frequently implicated in nosocomial infections thus, not surprisingly, being considered by the World Health Organization as a major global concern (World Health Organisation [WHO], 2014a, b).

*K. pneumoniae* is considered a commensal agent in horses and its clinical relevance and disease severity depend on the strain’s pathogenic potential (Turton et al., 2018). Additionally, horses have been shown to carry human associated MDR *K. pneumoniae* (Ewers et al., 2014) and ESBL-producing bacteria, but characterization of MDR isolates are still lacking for this particular species (Maddox et al., 2015), with only a few reports of ESBL-producing *K. pneumoniae* in horses available to date (Vo et al., 2007; Ewers et al., 2014; Walther et al., 2018).

Evidence is growing on the importance of shared MDR bacterial populations between humans and other animals such as companion animals (Ewers et al., 2014; Harrison et al., 2014), livestock (Dahms et al., 2015), or wildlife (Schaufler et al., 2018), including ESBL-producing strains (Weese et al., 2015). The misuse of antimicrobials in animals, has led the World Health Organization to develop in 2017 guidelines to minimize the administration of critical antibiotics to food producing animals. However, Portugal still remains one of the countries with the highest antimicrobial consumption per animal population (mg/PCU) in Europe (4th out of 30 countries; European Medicines Agency [EMA], 2018) This is worrying, since it imposes a selection pressure on pathogenic, commensal and environmental bacteria that could lead to the maintenance and recruitment of resistance genes (Bengtsson-Palme et al., 2017). It is known that bacteria isolated from horses share common genetic ground with other animals and human isolates (Schmiedel et al., 2014) and this supports the hypothesis that these animals are likely to have a particular role in antimicrobial resistance transmission. Therefore, efforts to better understand the role of horses as carriers of such pathogens are of major importance to public health.

In this study, we characterized an ESBL-producing *K. pneumoniae* sequence type (ST) 348 isolate from a horse by whole genome sequencing (WGS). Additionally, we compared this strain to ST348 human isolates previously obtained and characterized, in order to assess the genetic relationship between *K. pneumoniae* ST348 isolates from Portugal.

## Materials and Methods

### Sample Collection and Epidemiological Data

A 14-year-old Lusitano horse with signs of abdominal pain was referred to a referral equine hospital in Lisbon in June 2016 and diagnosed with a small intestinal inguinal hernia. Laparotomy was performed, and the incarcerated portion of non-viable small intestine was resected under general anesthesia. The horse had never been subjected to antimicrobial therapy before and was treated with penicillin at a dose of 22 000 UI/kg for 7 days and gentamicin at a dose of 6,6 mg/kg for 5 days post-surgery. The patient developed a surgical site infection of the abdominal wall that was detected on day 4 post-surgery. A swab sample was collected from the site and further streaked in Blood and MacConkey agar. After a 24 h incubation at 37°C, pure bacterial cultures were obtained, posteriorly identified as *K. pneumoniae* using API 20 *E* test (Biomérieux, Marcy-Étoile, France).

The Laboratory of Microbiology of the Faculty of Pharmacy of the University of Porto provided the seven ST348 human isolates (Rodrigues et al., 2016, 2017 and unpublished data).

### Antimicrobial Susceptibility Profile

The isolates susceptibility profile was determined with the Vitek 2 system, using the AST N344 card. The minimal inhibitory concentration (MIC) for ciprofloxacin was determined using *E*-test, following EUCAST breakpoint guidelines (The European Committee on Antimicrobial Susceptibility Testing [EUCAST], 2018).

### Whole Genome Sequencing and Data Analysis

Whole genome sequencing of all isolates was performed. DNA extraction was performed with the DNeasy UltraClean Microbial Kit (Qiagen, Hilden, Germany) and DNA concentration was measured with a Qubit 2.0 fluorometer (Life Technologies, Thermo Fisher Scientific, Waltham, MA, United States).

The DNA was then diluted to 0.2 ng/μl and 1 ng was used for the library preparation, using the Nextera XT Library Preparation kit (Illumina, CA, United States), according to the manufacturer’s protocol. DNA was sequenced using the Miseq (Illumina) generating 2 times 250-bp reads and *de novo* assembly was done by CLC Genomics Workbench v11.0 (Qiagen) after quality (Phred scores >30) and adapter trimming. Annotation was performed using the RAST server^1^. Resistome analysis was performed by uploading sequences to the CGE server^2^. Plasmid replicons were identified through Abricate v0.8-dev^3^, while the ST, *wzi*, K and O types and a virulence score, based on the presence or absence of different siderophores genes and a genotoxin gene, were determined through Kleborate v0.2.0^4^ and Kaptive v0.5.1^5^.

For MinION long-reads, base calling and demultiplexing was performed using Albacore v1.2.2 (ONT) and data quality was analyzed through Poretools v0.6.0 (Loman and Quinlan, 2014). Adapter trimming was attained through Porechop v0.2.3^6^. Hybrid assemblies of short- and long-reads were performed using Unicycler v0.4.1 (Wick et al., 2017). Bandage v0.8.1 (Wick et al., 2015) was used to visualize the assembly graphics. Easyfig was used to compare plasmids pK1 and pDA33141-217 (Sullivan et al., 2011). BRIG was used to align plasmid pK1 with the assemblies of the human strains (Alikhan et al., 2011).

The comparative phylogenetic analysis of the eight isolates was performed using a core genome (cg) MLST approach through Ridom SeqShere+ software (Ridom, Munster, Germany) and BacWGSTdb was used to perform a comparison with similar isolates (Ruan and Feng, 2016)^7^.

Amino-acid substitutions were investigated in the quinolone resistance determining regions (QRDR) of the *gyrA*, *gyrB*, *parC*, and *parE* genes.

## Results

### MDR *Klebsiella pneumoniae* ST348 in a Horse

The *K. pneumoniae* isolate obtained from the surgical site infection was resistant to the majority of the antimicrobials tested, which included different cephalosporins, fluoroquinolones and aminoglycosides (Table 1). WGS allowed the analysis of its resistome, revealing the presence of *bla*_CTX–M–15_, among several other resistance genes (Table 2). The complete sequence of a large plasmid was obtained through a combination of long- and short-read sequencing (∼190 kb), which carried several antimicrobial resistance genes (*bla*_CTX–M–15_, *bla*_TEM–1B_, *bla*_OXA–1_, *sul2*, *aph(6)-Id*, *aac(6′)-Ib-cr*, *catB4*, *tet*(A), *dfrA14*, *qnrB1*) and genes associated with metal tolerance (*ars* operon). Replicons from incompatibility groups IncFII and IncFIB were identified. A comparison between our plasmid pK1 and pDA33141-217, the closest plasmid in the Genbank database (CP029588; homology <99%, sequence cover <83%) was performed, and differences are mainly due to rearrangements in the region surrounding the *bla*_CTX–M–15_ gene. By using a BLAST approach, a similar plasmid was also found in the human strain 122.1, but not in the other human ST348 isolates.

**TABLE 1:** Susceptibility profile of the horse *K. pneumoniae* strain K.

**Phenotypic resistance**			**Breakpoint (mg/L)**	
**Antibiotic**	**MIC (mg/L)**		**Susceptible**	**Resistant**
Ampicillin	≥32	R	≤8	>8
Amoxicillin/clavulanic acid	≥32	R	≤8	>8
Piperacillin/tazobactam	32	R	≤8	>16
Cefuroxime	≥64	R	≤8	>8
Cefoxitin	≤4	S	≤8	>8
Cefotaxime	≥64	R	≤1	>2
Ceftazidime	32	R	≤1	>4
Imipenem	≤0.25	S	≤2	>4
Meropenem	≤0.25	S	≤2	>8
Gentamicin	≥16	R	≤2	>4
Tobramycin	≥16	R	≤2	>4
Ciprofloxacin	≥4	R	≤0,25	>0,5
Fosfomycin	≤16	S	≤32	>32
Nitrofurantoin	128	R	≤64	>64
Colistin	≤0.5	S	≤2	>2
Trimethoprim	≥16	R	≤2	>4
Sulfamethoxazole/trimethoprim	≥16	R	≤2	>4

**TABLE 2:** Antimicrobial resistance genes of the horse *K. pneumoniae* strain K.

				**Enzyme**			
**Antibiotic family**	**Resistance gene**	**% identity**	**Accession no.**	**Name**	**Function**
ß-Lactams	*bla*_TEM–1–B_	100.00	JF910132	TEM-1B	Penicillinase
	*bla*_SHV–11_	99.88	EF035557	SHV-11	Penicillinase
	*bla*_OXA–1_	100.00	J02967	OXA-1	ESBL
	*bla*_CTX–M–15_	100.00	DQ302097	CTX-M-15	ESBL
Aminoglycosides	*aac(6′)-Ib-cr*	100.00	DQ303918	AAC(6′)Ib-cr	Acetyltransferase
	*aph(3”)-Ib*	100.00	AF321551	APH(3”)-Ib	Phosphotransferase
	*aph(6)-Id*	100.00	M28829	APH(6)-Id	Phosphotransferase
	*aac(3)-IIa*	99.77	X51534	AAC(3)-IIa	Acetyltransferase
Fosfomycin	*fosA*	99.52	ACWO01000079	FosA	Mn2+ and K+-dependent glutathione S-transferase
Phenicols	*catB4*	100	EU935739	CatB4	Acetyltransferase
Quinolones	*oqxB*	99.08	EU370913	OqxB	Efflux
	*oqxA*	99.15	EU370913	OqxA	Efflux
	*aac(6′)-Ib-cr*	100.00	DQ303918	AAC(6′)Ib-cr	Acetyltransferase
	*qnrB1*	100	Q351241	QnrB1	Topoisomerase type II protection
Sulfonamides	*sul2*	100.00	GQ421466	Sul1	Efflux
Tetracyclines	*tet(A)*	100.00	AJ517790	TetA	Efflux
Trimethoprim	*dfrA14*	99.59	DQ388123	DfrA12	Dihydrofolate reductase

### Comparative Analysis With Human Isolates

A comparative analysis with other MDR *K. pneumoniae* ST348 isolated from humans in Portugal between 2012 and 2016 was performed to evaluate a possible genetic link. These human isolates were obtained as colonizers or causing infection in individuals in long-term health care facilities and hospitals, and produced CTX-M-15 and occasionally KPC-3 (Rodrigues et al., 2016, 2017; Table 3). As in the equine isolate, the same ST capsular type, KL62 (*wzi*94), and O1v1 antigen, were found. In addition, all isolates had similar content on ß-lactamases genes (*bla*_CTX–M–15_, *bla*_SHV–11_, *bla*_OXA–1_, and/or *bla*_TEM–1_), with differences for isolate Kp56 that had a carbapenemase gene, *bla*_KPC–3_, isolate 102-1 that lacked the *bla*_OXA–1_ gene, and isolate C1685 that lacked the *bla*_TEM–1B_ gene (Table 4). Additionally other antimicrobial resistance genes were also present in all isolates [*aac(6′)-Ib-cr*, *fosA*, *catB4*, *oqxA*, and *oqxB* (Table 4)]. All of them carried the FIB replicon, but variably the repFII or repFIA. The isolates were also analyzed concerning their virulence score, which, according to the score system provided by Kleborate, was 1 out of 5 for the isolates that contained the yersiniabactin gene, and 0 out of 5 for the only isolate in which this gene was not detected.

**TABLE 3:** Epidemiological and genetic features of the eight *K. pneumoniae* ST348 isolates studied.

	**C1682**	**C1685**	**C1741**	**Kp56**	**K107**	**102-1**	**122-1**	**K**
Sequence type (ST)	ST348	ST348	ST348	ST348	ST348	ST348	ST348	ST348
wzi	*wzi94*	*wzi94*	*wzi94*	*wzi94*	*wzi94*	*wzi94*	*wzi94*	*wzi94*
K antigen	KL62	KL62	KL62	KL62	KL62	KL62	KL62	KL62
O antigen	O1v1	O1v1	not identified	O1v1	O1v1	O1v1	O1v1	O1v1
Plasmid Inc., Group	IncFII, IncFIB	IncFIB	IncFII, IncFIB	IncFII, IncFIA, IncFIB	IncFII, IncFIB	IncFII, IncFIB	IncFIB	IncFII, IncFIB
ß-lactamases genes	CTX-M-15, SHV-11, OXA-1, TEM-1B	CTX-M-15, SHV-11, OXA-1	CTX-M-15, SHV-11, OXA-1, TEM-1B	KPC-3, CTX-M-15, SHV-11, OXA-1, TEM-1B	CTX-M-15, SHV-11, OXA-1, TEM-1B	CTX-M-15, SHV-11, TEM-1B	CTX-M-15, SHV-11, OXA-1, TEM-1B	CTX-M-15, SHV-11, OXA-1, TEM-1B
Source (Age/Gender)	Urine/UTI, Human (82/M)	Urine/UTI, Human (81/M)	Exudate/SSTI, Human (72/M)	Urine/UTI, Human (87/M)	Human (F)	Rectal swab, Human (F/89)	Rectal swab, Human (M/79)	SSI, Horse (M/14)
Date of isolation	14/08/12	18/08/12	20/09/12	03/10/14	07/10/15	07/01/16	07/01/16	16/06/16
Place of isolation	Community Lab, North, Portugal	Community Lab, North, Portugal	Community Lab, North, Portugal	Household resident, Oporto, Portugal	Hospital, Oporto, Portugal	LTCF resident, Oporto, Portugal	LTCF resident, Oporto, Portugal	Equine Hospital, Lisbon, Portugal

*F, female; M, male; SSI, Surgical Site Infection; SSTI, Skin and Soft Tissue Infection; and UTI, Urinary Tract Infection.*

**TABLE 4:** Resistance genes found in the eight *K. pneumoniae* ST348 isolates.

	**C1682**		**C1685**		**C1741**		**Kp56**		**K107**		**102-1**		**122-1**		**K**	
**Antibiotic family**	**Resistance gene**	**% identity**	**Resistance gene**	**% identity**	**Resistance gene**	**% identity**	**Resistance gene**	**% identity**	**Resistance gene**	**% identity**	**Resistance gene**	**% identity**	**Resistance gene**	**% identity**	**Resistance gene**	**% identity**
ß-Lactams	*bla*_*TEM–1–B*_	100.00	*bla*_*SHV–11*_	99.88	*bla*_*TEM–1–B*_	100.00	*bla*_*TEM–1–B*_	100.00	*bla*_*TEM–1–B*_	100.00	*bla*_*TEM–1–B*_	100.00	*bla*_*TEM–1–B*_	100.00	*bla*_*TEM–1–B*_	100.00
	*bla*_*SHV–11*_	99.88	*bla*_*OXA–1*_	100.00	*bla*_*SHV–11*_	99.88	*bla*_*SHV–11*_	99.88	*bla*_*SHV–11*_	99.88	*bla*_*SHV–11*_	99.88	*bla*_*SHV–11*_	99.88	*bla*_*SHV–11*_	99.88
	*bla*_*OXA–1*_	100.00	*bla*_*CTX–M–15*_	100.00	*bla*_*OXA–1*_	100.00	*bla*_*OXA–1*_	100.00	*bla*_*OXA–1*_	100.00	*bla*_*CTX–M–15*_	100.00	*bla*_*OXA–1*_	100.00	*bla*_*OXA–1*_	100.00
	*bla*_*CTX–M–15*_	100.00			*bla*_*CTX–M–15*_	100.00	*bla*_*CTX–M–15*_	100.00	*bla*_*CTX–M–15*_	100.00			*bla*_*CTX–M–15*_	100.00	*bla*_*CTX–M–15*_	100.00
							*blaKPC-3*	100.00								
Aminogly -cosides	*aac(6′)-Ib-cr*	100.00	*aac(6′)-Ib-cr*	100.00	*aac(6′)-Ib-cr*	100.00	*aac(6′)-Ib-cr*	100.00	*aac(6′)-Ib-cr*	100.00	*aac(6′)-Ib-cr*	100.00	*aac(6′)-Ib-cr*	100.00	*aac(6′)-Ib-cr*	100.00
	*aph(3″)-Ib*	100.00			*aph(3″)-Ib*	100.00	*aph(3″)-Ib*	100.00	*aph(3″)-Ib*	100.00	*aph(3″)-Ib*		*aph(3″)-Ib*	100.00	*aph(3″)-Ib*	100.00
	*aph(6)-Id*	100.00			*aph(6)-Id*	100.00	*aph(6)-Id*	100.00	*aph(6)-Id*	100.00	*aph(6)-Id*	100.00	*aph(6)-Id*	100.00	*aph(6)-Id*	100.00
	*aac(3)-IIa*	100.00	*aac(3)-IIa*	100.00	*aac(3)-IIa*	100.00	*aac(3)-IIa*	100.00	*aac(3)-IIa*	100.00			*aac(3)-IIa*	100.00	*aac(3)-IIa*	99.77
Fosfomycin	*fosA*	99.52	*fosA*	99.52	*fosA*	99.52	*fosA*	99.52	*fosA*	99.52	*fosA*	99.52	*fosA*	99.52	*fosA*	99.52
Phenicols	*catB4*	100.00	*catB4*	100.00	*catB4*	100.00	*catB4*	100.00	*catB4*	100.00	*catB4*	100	*catB4*	100	*catB4*	100
Quinolones	*oqxB*	99.08	*oqxB*	99.08	*oqxB*	99.08	*oqxB*	99.08	*oqxB*	99.08	*oqxB*	99.08	*oqxB*	99.08	*oqxB*	99.08
	*oqxA*	99.15	*oqxA*	99.15	*oqxA*	99.15	*oqxA*	99.15	*oqxA*	99.15	*oqxA*	99.15	*oqxA*	99.15	*oqxA*	99.15
	*aac(6′)-Ib-cr*	100.00	*aac(6′)-Ib-cr*	100.00	*aac(6′)-Ib-cr*	100.00	*aac(6′)-Ib-cr*	100.00	*aac(6′)-Ib-cr*	100.00	*aac(6′)-Ib-cr*	100.00	*aac(6′)-Ib-cr*	100.00	*aac(6′)-Ib-cr*	100.00
	*qnrB1*	99.07			*qnrB1*	99.07	*qnrB1*	100.00	*qnrB1*	100.00	*qnrB1*	100.00	*qnrB1*	100.00	*qnrB1*	100
Sulfonamides													*sul1*	100.00		
	*sul2*	100.00			*sul2*	100.00	*sul2*	100.00	*sul2*	100.00	*sul2*	100.00	*sul2*	100.00	*sul2*	100.00
Tetracyclines	*tet(A)*	100.00			*tet(A)*	100.00	*tet(A)*	100.00	*tet(A)*	100.00	*tet(A)*	100.00	*tet(A)*	100.00	*tet(A)*	100.00
Trimethoprim	*dfrA14*	99.59			*dfrA14*	99.59	*dfrA14*	99.59	*dfrA14*	99.59	*dfrA14*	99.59	*dfrA14*	99.59	*dfrA14*	99.59

**FIGURE 1:**
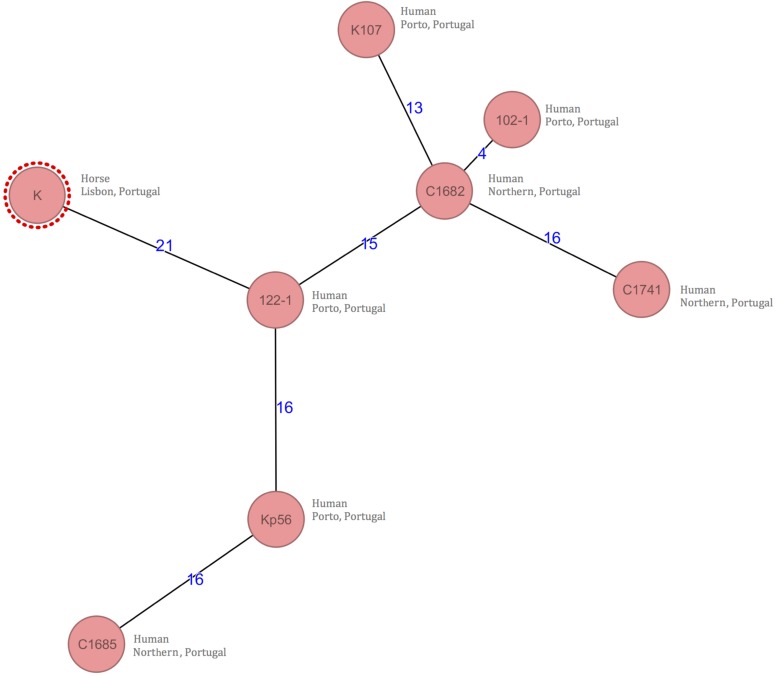
Minimum-spanning tree based on cgMLST allelic profiles of 8 *K. pneumoniae* genomes obtained through WGS. Each circle represents an allelic profile based on sequence analysis. The numbers on the connecting lines illustrate the numbers of target genes with differing alleles.

The clonal relatedness was evaluated using a gene-by-gene typing method (core genome Multi-Locus Sequence Typing [cgMLST]). Allele differences between the isolates ranged from 4 to 21 in a total of 2358 genes. There were 21 allele differences between the horse isolate (K) and the closest human colonizing isolate (isolate 122-1) obtained from a long term-care facility resident (Figure 1). Using the BacWGSTdb, 56 allele differences between the horse isolate (K) with the closest isolate deposited in the database were detected. Again, this isolate corresponded to a human strain from a respiratory infection in Portugal in 2013.

### *Klebsiella pneumoniae* ST348 QRDR

Our collection of *K. pneumoniae* ST348 showed no mutations in the QRDR regions, except for two isolates (C1685 and Kp56) that presented a mutation in codon 83 of the *gyrA* gene. MIC results to ciprofloxacin ranged from 0.75 mg/L to 8 mg/L. One isolate (K107) presented high resistance to ciprofloxacin, with an MIC of >32 mg/L.

## Discussion

### MDR *Klebsiella pneumoniae* ST348 in a Horse

According to the Centers for Disease Control and Prevention (CDC), complications or infections secondary to either device implantation or surgery are referred to as hospital-acquired infections (Boev and Kiss, 2017). Acquisition of infections within the hospital are silent threats, especially when the source remains unknown (Walther et al., 2018). This horse developed a surgical site infection due to a MDR *K. pneumoniae* during hospitalization, which was therefore considered a hospital-acquired infection. We do not know if the contamination source was exogenous (i.e., surgical personnel) or intrinsic (i.e., horse’s skin) and if it was developed during, or after surgery. However, a recent study showed that a positive intra-operative culture was not a predictor of SSI; and even when a SSI did occur, it may be due to a different bacterial isolate (Isgren et al., 2019). The occurrence of ESBL-producing bacteria in horses (Johns et al., 2012; Apostolakos et al., 2017), including isolates containing the *bla*_CTX–M–15_ gene (Leigue et al., 2018), has been shown in previous studies. The challenge of treating such infections can be overcome by the use of antiseptics (Southwood, 2009) without the need for treatment with antimicrobials. This was the case in this patient, where the infected wound was successfully treated with lavages using a saline solution containing 0.05% of chlorhexidine, performed twice a day for 1 week (Wilkins and Unverdorben, 2013).

### *K. pneumoniae* ST348 Clones

The isolate obtained from the wound was identified as a *K. pneumoniae* ST348, a ST not previously reported in animals. In addition, it has rarely been identified in international collections and databases of MDR *K. pneumoniae* isolates from humans (Baraniak et al., 2013; Breurec et al., 2013; Ruan and Feng, 2016) or in other hosts (Holt et al., 2015), and there is still scarce surveillance data on different non-human niches. Indeed, most reports of ST348 *K. pneumoniae* in humans are from the Northern region of Portugal, where this lineage seems to be circulating since at least 2012 as a CTX-M-15 producer that subsequently acquired KPC-3. The isolates were obtained from community-based patients or residents in long-term care facilities with previous contact to different hospitals, suggesting a more extended dissemination in the country (Rodrigues et al., 2014, 2016, 2017; Vubil et al., 2016).

Several studies have associated quinolone resistance to a higher fitness cost and the establishment of major and minor clones in different bacterial species (Marcusson et al., 2009; Fuzi, 2016). Tóth et al. (2014) have related a high number of amino-acid substitutions in QRDR with a better fitness *in vitro* and *in vivo* (e.g., major clones) in *K. pneumoniae* species. Results from this study are in accordance with this, as our isolates present no mutations, except for two isolates (C1685 and Kp56) that presented only one mutation. Although these ST348 isolates are CTX-M-15 producers, we hypothesized that this clone is also a minor clone that suffered large fitness cost with the introduction of fluoroquinolones but still acquired alternative mechanisms of resistance {both efflux mediated resistance [*aac(6′)-Ib-cr*, *oqxA*, and *oqxB* genes] and *qnr* determinants}, thus being a minor clone with a better fitness adaptation. The MIC results of ciprofloxacin also support this hypothesis as our isolates present MIC’s ranging from 0.75 mg/L to 4 mg/L that are lower than those associated with major clones (Tóth et al., 2014) with two exceptions for isolates C1741 (MIC 8 mg/L), and K107 (MIC > 32 mg/L), showing that this clone has also a better adaptation to these drugs.

### Comparative Analysis With Human Isolates

A cgMLST analysis, comparing the horse and human isolates, was performed to detect a possible genetic link that could provide evidence for the source of the *K. pneumoniae* strain (Figure 1). Recent cut-offs advocate that clonal relatedness is accepted for the *K. pneumoniae* isolates when allele difference are less than 10 (Schürch et al., 2018); using this cutoff we could assume that there is no genetic link between the horse and human isolates. Despite that, other authors have interpreted these cutoffs from a different perspective as they advocate that 16 to 21 allele differences between isolates from carriers from different species suggest that transmission has occurred (Adler et al., 2017; Lifshitz et al., 2018). Moreover, most of the few studies that define cutoffs for cgMLST analysis are based on human isolates only (Schürch et al., 2018) and do not take into account interspecies transmission.

A direct clinical epidemiological link between the horse isolate and the human isolates could not be found except for the year of isolation: none of the horses’ tutors had contact with hospitals or long-term care facilities in the Northern part of Portugal, where the horse stable was located, and none of the veterinarians and caretakers had been previously infected or known to be colonized with an ESBL-*K. pneumoniae* strain (although active surveillance was not routinely performed). Furthermore, it was not possible to establish the previous colonization of the horse, as samples are usually not collected prior to emergency surgeries.

Taking into consideration the similarities between the horse and human isolates, one can consider the possibility of a silent dissemination of *K. pneumoniae* ST348 amongst humans and horses in Portugal. A direct human-to-animal transmission or a common source of infection cannot be ruled out, especially since active surveillance of MDR pathogens of people in contact with the horse was not performed. The low virulence presented by these isolates and therefore the improbability of the isolation of this particular ST of *K. pneumoniae*, also supports the hypothesis of a silent dissemination. This is also enhanced by the results revealed by the cgMLST analysis performed with BacWGSTdb, since all the *K. pneumoniae* ST348 strains deposited in this database were human strains, the majority from Portugal.

Horses have different uses in today’s society, which include sports, leisure, production and consumption. It is very likely that the lack of active surveillance and characterization of antimicrobial resistant bacteria in these animals, and the proximity to man is contributing to the spread and evolution of these MDR strains. This report suggests that some *K. pneumoniae* clones, as the ST348, can carry critical resistance genes and disseminate them in different populations, despite their low virulence.

## Ethics Statement

The animal was cared for according to the rules established by the EU (Directive 2010/63/EC) and national (DL 113/2013) legislation and by the competent authority (Direção Geral de Alimentação e Veterinária, DGAV, www.dgv.min-agricultura.pt/portal/page/portal/DGV) in Portugal. Only noninvasive samples were collected during routine procedures with consent of the owner, and no ethics committee approval was needed. Trained veterinarians obtained the samples following standard routine procedures. No animal experiment was performed in the scope of this research. Verbal informed consent was obtained from the owner after being informed about the study.

## Author Contributions

FT and NC performed the experiments, analyzed the data, and wrote the manuscript. CC participated in the experiments. EC participated in the experiments and helped to analyze the data. TR and MM being clinicians, were responsible for the clinical case. LT contributed to the analysis and interpretation of data and helped to draft the manuscript. ÂN and LP participated in the experiments and helped to draft and revise the manuscript. JR contributed to the analysis and interpretation of data and helped to draft and revise the manuscript. LL and MO conceived the study and participated in its coordination, helped to draft the manuscript, and supervised the study throughout.

## Conflict of Interest Statement

JR consults for IDbyDNA. All authors have submitted the ICMJE Form for Disclosure of Potential Conflicts of Interest. Conflicts that the editors consider relevant to the content of the manuscript have been disclosed.

## Footnotes

1 rast.nmpdr.org

2 https://cge.cbs.dtu.dk

3 https://github.com/tseemann/abricate

4 https://github.com/katholt/Kleborate

5 https://github.com/katholt/Kaptive

6 https://github.com/rrwick/Porechop

7 http://bacdb.org/BacWGSTdb/
